# Evaluation of Microalgae as Immunostimulants and Recombinant Vaccines for Diseases Prevention and Control in Aquaculture

**DOI:** 10.3389/fbioe.2020.590431

**Published:** 2020-11-16

**Authors:** Ke Ma, Qiuwen Bao, Yue Wu, Siwei Chen, Shuxin Zhao, Haizhen Wu, Jianhua Fan

**Affiliations:** ^1^State Key Laboratory of Bioreactor Engineering, East China University of Science and Technology, Shanghai, China; ^2^Department of Applied Biology, East China University of Science and Technology, Shanghai, China

**Keywords:** aquaculture industry, microalgae, immunostimulant, oral vaccine, polymicrobial infections

## Abstract

Microalgae are often used as nutritional supplements for aquatic animals and are widely used in the aquaculture industry, providing direct or indirect nutrients for many aquatic animals. Microalgae are abundant in nature, of high nutritional value, and some of them are non-toxic and rich in antioxidants so that they can be explored as a medicinal carrier for human or animals. Natural wild-type microalgae can be adopted as an immunostimulant to enhance non-specific immune response and improve growth performance, among which *Haematococcus pluvialis, Arthrospira (Spirulina) platensis, and Chlorella* spp. are commonly used. At present, there have been some successful cases of using microalgae to develop oral vaccines in the aquaculture industry. Researchers usually develop recombinant vaccines based on *Chlamydomonas reinhardtii, Dunaliella salina*, and cyanobacteria. Among them, in the genetic modification of eukaryotic microalgae, many examples are expressing antigen genes in chloroplasts. They are all used for the prevention and control of single infectious diseases and most of them are resistant to shrimp virus infection. However, there is still no effective strategy targeting polymicrobial infections and few commercial vaccines are available. Although several species of microalgae are widely developed in the aquaculture industry, many of them have not yet established an effective and mature genetic manipulation system. This article systematically analyzes and discusses the above problems to provide ideas for the future development of highly effective microalgae recombinant oral vaccines.

## Introduction

The aquaculture industry has developed rapidly in recent years and provided more fish products for human food supply than that did capture fisheries for the first time in 2014 (Pauly and Zeller, 2017). According to the model proposed by Kobayashi et al. (2015), in face of the rapid expansion of global fish demand and the relatively stable capture fisheries, aquaculture is expected to fill the widening gap between food supply and demand, especially in Asia. By 2030, aquaculture will provide about 62% of fish for human consumption. Moreover, after 2030, aquaculture is likely to continue to dominate the future global fish supply and grow sustainably.

However, the outbreak of diseases will bring severe losses to the aquaculture industry economically. Severe diseases in aquaculture are mostly caused by viruses and various protists, as well as bacteria (Lafferty et al., 2015). Although some bacterial diseases can be treated by several vaccines and antibiotics effectively, others cannot be solved by antibiotics. With the abuse of antibiotics, the amount of antibiotic-resistant bacteria and the risk of remaining antibiotics and antibiotic-resistant bacteria in aquatic products transferring to the human body have been increasing (Costa et al., 2015), arising great attention globally, which leads to the development of new antibiotic alternatives to control the diseases. Recently, the developed vaccines using microalgae as carriers have been increasingly applied to the aquaculture industry, which provides new ideas for diseases prevention and control in aquaculture.

**FIGURE 1:**
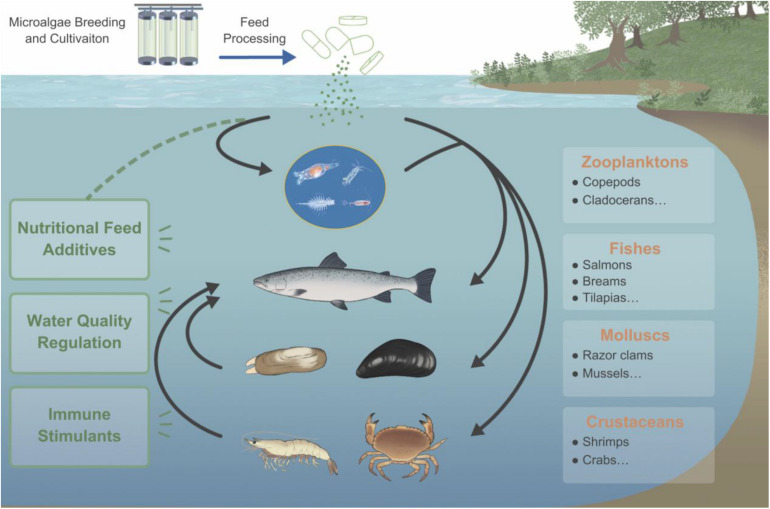
The importance of microalgae in the aquaculture industry. Microalgae can provide direct or indirect nutrition for aquatic animals and can be immune stimulants for diseases control, also play an important role in regulation of water quality.

As an essential part of aquatic ecosystems like oceans and lakes, microalgae is of great significance to the aquatic environment, the health of aquatic animals, and the balance of the ecosystem (Figure 1). The cells of microalgae are rich in active nutrients such as proteins, polyunsaturated fatty acids (PUFAs), polysaccharides, and essential amino acids, which can promote the growth of fish, shrimp, crab, and shellfish. Therefore, they can be used as a basic feed for fish and other economic aquatic animals directly or indirectly (Yaakob et al., 2014). In addition, microalgae also play an important role in regulating and judging the quality of aquaculture water. For example, algae cells can absorb nutrients such as nitrogen and phosphorus to improve the water quality and maintain a good dynamic balance, thereby enhancing the disease resistance of aquatic animals and improving the survival rate (Taelman et al., 2013). Due to the increasing importance of microalgae in aquaculture, this article reviews the aspects of microalgae in the prevention and control of diseases in aquaculture. Before conceiving and writing this review, we had searched the literature thoroughly, whatever in the past few years or decades. There are roughly hundreds of papers in the field of algae application in aquaculture. However, there is few of systematic summary of the existing research conclusions, especially few about evaluation of microalgae as immunostimulants and recombinant vaccines for disease prevention and control in aquaculture. Charoonnart et al. (2018) reviewed the genetic engineering of microalgae to produce therapeutic proteins and biomolecules against aquaculture diseases, in which some research cases were listed and summarized. Abidin et al. (2020) focused on the potential and application of genetically modified microalgae in aquaculture, and evaluated the feasibility of microalgae as a vaccine carrier from a technical perspective. This article systematically analyzes and discusses the above problems, including some examples of commercial application, in order to provide ideas for the future development of highly effective microalgae recombinant oral vaccines.

## The Importance of Microalgae as Feed for Aquaculture Industry

For a long time, microalgae have provided direct or indirect nutrition to the early stages of growth of many aquaculture fish, shellfish, and invertebrates (Priyadarshani and Rath, 2012). In the larval stages of mollusks, echinoderms, crustaceans, and some fish, the feeding method is usually filter feeding, during which microalgae are the source of nutrients (Hemaiswarya et al., 2010; Kaparapu, 2018). Tredici et al. (2009) have reviewed the cultivation patterns of microalgae used for feeding. They introduced the development of microalgae biotechnology, especially the new culture techniques, and focused on the practical and potential applications of algae in the nutrition of aquatic animals, which can take a dominant position in this ecosystem. Shields and Lupatsch (2012) have summarized the current state of algae use in aquaculture and developments in algal biomass as an ingredient in formulated animal feeds. Microalgae provide an important direct or indirect feed source for the early developmental stages of many aquatic species, and traditionally, in hatcheries for aquatic animals, microalgae farming is large-scale, generally in outdoor ponds or large pools. However, in intensive aquaculture hatcheries, microalgae breeding is usually carried out in specialized bioreactors, managing different algae species regularly through artificial or automated means.

Microalgae are generally single cells of size from several to dozens of micrometers. The individual size and structural function of microalgae as well as the feeding structure and digestive function of aquatic animals exist differences. Thus different aquatic animals require nutrients from respective types of feeding microalgae at different growth stages. For example, microalgae rich in eicosapentaenoic acid (EPA) or docosahexaenoic acid (DHA) (*Chaetoceros calcitrans*, *Isochrysis galbana*, etc.) are usually provided for marine mollusks in the early development stage (Ran et al., 2020). Microalgae that are inseparable from the whole-life growth and development of shellfish, such as *Tetraselmis* spp., *Thalassiosira pseudodonana*, *and C. calcitrans*, are usually supplied for crustaceans. *Nannochloropsis* spp. are mainly used in the culture of rotifers. *Dunaliella salina* is often used for pigmentation of aquatic animals (Borowitzka, 1997). As the number and species of aquaculture animals increase, so does the demand for suitable microalgae in the aquaculture industry. At the growth and metamorphic development stage of fish, shrimp, shellfish, and crab larvae, the mixed feeding of microalgae and animal-based natural bait would lead to better results (Shields and Lupatsch, 2012).

At present, there are dozens of kinds of bait microalgae that can be popularly applied in large-scale seedling production all over the world. Microalgae are the major source of food for zooplankton and small-size fish, and subsequently a valuable source of vital nutrients as fodder for fish in the upper echelons of the food chain, microalgae-based feeds offer promising food sources for sustainable aquaculture industry (Yarnold et al., 2019). Shah et al. (2017) reviewed the latest progress of microalgae as a supplement or feed additive to replace fishmeal and fish oil in aquaculture. According to the nutrient requirements of fish, good selection of microalgae species can improve its conversion rate in fish body, thus studying the nutrient composition of different microalgae is necessary to support aquaculture. In addition, the safety and regulatory issues of microalgae feed applications also need to be considered. Also, the cost of high-quality algal biomass is considerably higher than fishmeal or grain-based feed components, possibly limiting its large-scale application. It is worth noting that the algal biomass applied to the aquaculture feed industry is mainly in the form of pasting, thus the cost could be greatly reduced in this form (Raja et al., 2018). Of course, the algae species that are being developed and commonly used are usually non-toxic, harmless, nutritious, and easy to grow on high density at large scale.

Although the value of bait microalgae is increasingly recognized, in order to meet the development needs of aquatic animal larvae, further screening and directional cultivation of microalgae is indispensable. Moreover, it is urgent to establish the nutritional value evaluation system of microalgae bait and study new breeding methods and technologies to improve the nutritional value of bait microalgae. In conclusion, microalgae are absolute highly-demanded products in the field of aquaculture, with unique advantages and broad application prospects.

## Microalgae Can Be Excellent Immunostimulants or Antioxidants

Natural microalgae are rich in natural products, pigments, proteins, vitamins, PUFAs, and polysaccharide derivatives, and are the natural feed of aquatic animals, so it has inherent advantages to use them to develop microalgae additives. Bioactive substances from microalgae have natural antibacterial activity, which could eventually kill or inhibit the growth of pathogenic bacteria. Some polysaccharides were reported to increase the phagocytic capacity of macrophages and the gene expression level of pro-inflammatory cytokines, thereby activating natural immune response (Mohan et al., 2019). In addition, some PUFAs have unique regulatory effects on growth performance, membrane permeability, enzyme activity, immune function, etc. Yaakob et al. (2014) reviewed the contribution of microalgae in the nutritional requirements of aquatic feed. The nutrients contained in different microalgae are also different, and these main biomass ingredients in aquatic animals can play a role in enhancing the immune system and improving the function of anti-infection. From the perspective of the application status of microalgae feed in fish nutrition, microalgae also play a positive role in improving the growth performance, disease resistance, and skin color of edible fish (Roy and Pal, 2014; Molino et al., 2018). Because the nutrient characteristics of different algae are different, they can be used differently in aquaculture, so it is necessary to study the economic benefits of microalgae as feed additives to different aquaculture animals.

### Haematococcus pluvialis

There are lots of studies that used pigment-rich algae species as feeding additives. Sheikhzadeh et al. (2012) found that astaxanthin-rich *Haematococcus pluvialis* was added to fish feed in different proportions. Especially when the ratio is 0.3%, it promotes the physiological and metabolic functions of rainbow trout (*Oncorhynchus mykiss*), and improves the antioxidant activity of fish tissue effectively. Li et al. (2014) compared the effects of adding astaxanthin and *H. pluvialis* in feed on growth performance, antioxidant activity, and immune response of large yellow croakers (*Pseudosciaena crocea*). They found that dietary supplementation of both astaxanthin and *H. pluvialis* can improve the growth performance of large yellow croakers, whereas the latter is more effective. Furthermore, when the additive proportion of *H. pluvialis* is 0.28–0.56%, it can significantly improve the blood indices of large yellow croakers, and improve antioxidant and immune capacity. Astaxanthin can enhance the salinity stress tolerance, and salinity stress test is a widely used criterion to predict the health status of shrimp. Xie et al. (2018) found that dietary supplementation of 0.33–0.67% *H. pluvialis* improved the survival rate of white shrimp *Litopenaeus vannamei* under the salinity stress. One of the lipid peroxidation products malondialdehyde (MDA) and mRNA expression of superoxide dismutase (SOD) and glutathione peroxidase (GSH-PX) decreased in white shrimp larval livers after salinity stress, whereas total antioxidant capacity (T-AOC) increased. In addition, they demonstrated that the addition of *H. pluvialis* is mainly enhanced by the regulation of the NF-κB pathway, which indicated that the astaxanthin might improve the anti-inflammation and immune property. Thus, the intake of these substances or algae powder can improve the antioxidant performance as well as has a beneficial effect on innate immunity. Notably, there is a significant economic barrier in natural algal powder application, as that synthetic astaxanthin produced from petrochemicals is considerably cheaper than natural sources from *H. pluvialis*, and therefore the synthetic form dominates in the aquaculture industry (Shah et al., 2016).

### *Arthrospira platensis* (*Spirulina*)

*Arthrospira platensis* (also known as *Spirulina*) is a kind of cyanobacteria that has become increasingly popular worldwide as a dietary supplement, one of the most nutritious foods known to human, and is often used as a feed additive in aquaculture. Mahmoud et al. (2018) studied the effect of dietary adding *A. platensis* on nile tilapia (*Oreochromis niloticus*) growth performance, immune response, and the oxidation resistance. They found that dietary supplementation of *A. platensis* has no effect on the growth performance of tilapia, but significantly improves the antioxidant capacity and ability to resist the infection of *Pseudomonas fluorescens*, especially when adding proportion is 1%. Radhakrishnan et al. (2014) used *A. platensis* and Chlorella *vulgaris* instead of fishmeal to feed *Macrobrachium rosenbergii*. In all feeding groups, 50% of the fishmeal replacement group showed better non-enzymatic antioxidant activity, while no significant increases were shown in enzymatic antioxidant performance. Macias-Sancho et al. (2014) added different proportions of *A. platensis* to the fishmeal of the white shrimp *L. vannamei*. With the increasing proportion, the number of granular hemocytes increased significantly and the reduction of hemolymph cellular apoptosis prevented the early stage of virus infection. Yeganeh et al. (2015) evaluated the effect of dietary addition of *A. platensis* on the immune function of rainbow trout (*O. mykiss*). They found the immune response increases when supplementation ratio at 7.5 and 10%, and hematology and serum biochemistry related indicators of rainbow trout improved. Adel et al. (2016) have explored the effects of adding *A. platensis* diet on humoral immunity and mucosal immune response, and disease resistance of great sturgeon (*Huso huso*). They found that adding 5 or 10% of *A. platensis* significantly increased serum IgM, and the activity of lysozyme and the total protein content on the mucosa increased, thereby improving the ability of the great sturgeon to resist various pathogens. Chen et al. (2016) investigated the effects of *A. platensis* dried powder on the immune response of white shrimp *L. vannamei. A. platensis* can induce degranulation of shrimp hemocytes and increase oxidative stress response *in vitro* experiments. The 3 and 6% *A. platensis* diet feeds significantly stimulated shrimp innate immunity by increasing the expression of pattern recognition proteins (PRPs) like LGBP, as well as by increasing lysozyme activity, phagocytic activity, and resistance against *Vibrio alginolyticus*. Yu et al. (2018) found that feeding coral trout (*Plectropomus leopardus*) diets containing *A. platensis* (especially 10%) significantly improved their growth performance and the antioxidant status of livers, and enhanced the immune ability and resistance against *Vibrio harveyi*. Promya and Chitmanat (2011) found that adding 5% *A. platensis* or *Cladophora* spp. to the diet increased the number of red and white blood cells of African sharptooth catfish, and improved lysozyme activity in sera. Raji et al. (2018) explored fishmeal replacement with *A. platensis* and *C. vulgaris* in African catfish *Clarias gariepinus*, which have significantly improved the activity of CAT and the number of white blood cells. This indicates that the supplementation of these microalgae can stimulate immune response and improve the ability to fight infection.

### *Chlorella* spp.

From the view of nutrition, *C. vulgaris* is rich in nutrients, containing 61.6% proteins, 12.5% fat, 13.7% carbohydrates, trace elements, various vitamins, and minerals, and is generally used as growth promoter and immunopotentiator (Ahmad et al., 2018). Khani et al. (2017) have studied the effect of *C. vulgaris* on immunological parameters of koi carp *Cyprinus carpio*. They found that the addition of 5% dry *C. vulgaris* powder to koi carp diet can make fish with the highest level of IgM and lysozyme activity, resulting in resistance to both unsuitable environmental conditions and outbreaks of infectious diseases. Xu et al. (2014) indicated that when feeding gibel carp *Carassius auratus gibelio* with *C. vulgaris* as the dietary additive, with the increase of dietary proportion (0.4–2.0%), the relevant immunological parameters (SOD, POD, LZM, etc.) of gibel carp also showed an increasing trend. Zhang et al. (2014) also studied the effect of feeding *C. vulgaris* on the immune status of gibel carp (*C. a. gibelio*) and analyzed the relevant immunological parameters, reporting that *C. vulgaris* could increase the level of IgM and IgD, interleukin-22, and chemokine(C-C motif) ligand 5 in some tissues. Zahran and Risha (2014) and Zahran et al. (2018) found that dietary supplementing *C. vulgaris* at 10% could protect nile tilapia *O. niloticus* against arsenic-induced immunotoxicity and oxidative stress. Maliwat et al. (2017) added *C. vulgaris* to the giant freshwater prawn *M. rosenbergii*, which improved the prophenol oxidase activity and the total amount of hematocytes of *M. rosenbergii* postlarvae and could enhance the larval survival to *Aeromonas hydrophila* infection, especially at the supplementation ratio of 6%. Galal et al. (2018) found that *C. vulgaris* dietary supplementation could protect nile tilapia *O. niloticus* from being exposed to sub-lethal concentrations of penoxsulam herbicide and improve its anti-infective capacity against *Aeromonus sobria*.

### Other Algae Species

Research by Das et al. (2013) has shown that long-term dietary supplementation of dry *Microcystis aeruginosa* can significantly improve the immunity and the survival rate of Indian major carp *Labeo rohita*. However, *M. aeruginosa* can secrete some toxins that threaten health. They found that when feeding *L. rohita* in a ratio of 0.1%, it could significantly stimulate the immune system, improving the defense against *A. hydrophila.* Lyons et al. (2017) found that controlling the intestinal microbial community by dietary supplementation of 5% *Schizochytrium limacinum* might be a promising method to improve the intestinal health and nutrient utilization of rainbow trout *O. mykiss*. Nevertheless, it should be highlighted that the heterotrophic protist *S. limacinum* is technically not an algae, although it is often (incorrectly) named as such in publications and marketing (Leyland et al., 2017). What is noteworthy is that *S. limacinum* is rich in PUFAs and can be applied to the aquaculture industry. Salvesen et al. (1999) fed juvenile turbot *Scophthalmus maximus* L. with microalgae *I. galbana* matured water and found that such green water could improve the survival rate of juvenile turbot as well as accelerate its growth performance and ability to inhibit the proliferation of bacteria. Molina-Cárdenas and Sánchez-Saavedra (2017) found that six benthic diatom species had inhibitory effects on the growth of three common pathogens (*V. alginolyticus*, *Vibrio campbellii*, and *V. harveyi*), which could infect mollusks, shellfish, and fish. Moreover, many reports about the antibacterial properties of microalgae have pointed out that some metabolites secreted by microalgae can prevent pathogenic microorganisms from infecting their host. For example, marine algal polysaccharides play an antiviral role by inhibiting the adhesion of viruses (Ahmadi et al., 2015). And dietary supplementation of marine-derived polysaccharides can improve the growth, immune response, and disease resistance of aquatic animals (Mohan et al., 2019).

The researches mentioned above have shown that these microalgae can successfully enhance the innate immune function of the host, improve the antioxidant capacity, or reduce the infection of the pathogens to a certain extent. Therefore, some wild-type microalgae can act as effective immunostimulants. In addition, some studies have shown that microalgae can be used as a platform for protein production and drug delivery through genetic engineering. Although microalgae and its extracts have significant ability to prevent and control aquatic animal bacterial diseases, the antibacterial mechanism is still unclear, which should be strengthened in the future.

## Genetic Modified Microalgae Has Great Potential in Disease Control

### The Potential for Microalgae as Bioreactors to Produce Vaccines or Other Products

The development of microalgae cells as vaccine has long attracted the attention of scientists. In human diseases, the first reported antigen expressed using microalgae as a carrier was the capsid protein VP1 of foot-and-mouth disease virus (FMDV). Researchers assembled the gene of the cholera toxin B subunit (CTB) and the gene encoding VP1 into a chloroplast expression vector of *Chlamydomonas reinhardtii*, and finally integrated the chloroplast genome for expression. The expression level of the fusion protein can reach 3% of the total soluble protein (TSP) after detection (Sun et al., 2003). So far, researchers have used microalgae as a host to express relevant antigens of human diseases into preclinical trials (Rosales-Mendoza, 2016b). For example, in the field of parasitic and infectious diseases, multiple antigens such as Pfs25/28, D2-CTB, and Pfs25/28, D2-CTB, have been expressed in microalgal cells, respectively, against *Plasmodium falciparum*, *Staphylococcus aureus*, human papilloma virus, hepatitis B virus, and HIV (Specht and Mayfield, 2014; Rasala and Mayfield, 2015; Yan et al., 2016). In the field of non-infective diseases, genetically engineered microalgae have been developed for diseases such as type I diabetes, atherosclerosis, hypertension, allergies, and tumors (Rasala and Mayfield, 2015; Rosales-Mendoza, 2016b). There are also many successful reports on the use of microalgal cells to develop animal pathogenic vaccines. A number of microalgal recombinant subunit vaccines have been developed against classical swine fever virus, FMDV, etc. (Specht and Mayfield, 2014; Rasala and Mayfield, 2015; Rosales-Mendoza, 2016a; Yan et al., 2016). Throughout these studies, *C. reinhardtii* was used as a mature host system to express antigenic proteins, and the expression of foreign proteins is mainly between 0.1 and 5% TSP (Specht and Mayfield, 2014). Although *C. reinhardtii* is a model organism, due to random integration event, nuclear transformation is often accompanied by transgenic silencing, while transgenes inserted into the chloroplast genome by homologous recombination are not silenced and offer a platform for the production of recombinant proteins. Hence, the chloroplast homologous recombination is the main expression method, and nuclear transformation is randomly integrated as the auxiliary. Researchers also explored *Chlorella ellipsoidea* as an expression host to heterologously express and purify the rabbit defensin (NP-1). They obtained a small molecule defensin peptide NP-1 with high bacteriostatic activity, which also made it possible to produce such antibacterial peptides on a large scale with algae (Bai et al., 2013). At present, the application of microalgae and microalgae products in the pharmaceutical industry is attracting more and more attention. As the biotechnology for algae active substance research gradually matures, the use of microalgae for research and development of new drugs or vaccines has great potential.

### Prevention of Aquaculture Diseases by Microalgae Vaccine

It is worth noting that the microalgae oral vaccines have been designed and developed for specific pathogens in the field of aquaculture, and their immunoprotection has been explored. In order to prove the feasibility of the microalgae can be utilized as a oral delivery vector, Kwon et al. (2019) fed zebrafish (Danio rerio) on a diet of green fluorescent protein (GFP)-expressed *C. reinhardtii*, clear fluorescent signals in the intestinal tract can be detected by laser confocal image and immunostain, and GPF can also be detected in zebrafish serum, which indicates that the orally delivered proteins were protected until they were released in the gut. The research demonstrated the ability of *C. reinhardtii* as an oral delivery platform for recombinant bioactive proteins. The researchers Siripornadulsil et al. (2007) expressed the antigen protein p57 of *Renibacterium salmoninarum* in *C. reinhardtii*, and studied the effects of *in vivo* algae cell soaking and algae meal addition on the immune response induced by iris fish larvae. Their results showed that antibody production can be detected by soaking for 2 h or by adding 4% of the microalgae feed. Moreover, it was confirmed that the transgenic microalgae oral administration of this antigen presentation method could completely induce antibody production in blood, skin, epithelial tissue, and mucosa. Michelet et al. (2011) successfully genetically modified *C. reinhardtii* and efficiently expressed the two antigenic proteins AcrV and VapA of *Aeromonas salmonicida* in chloroplasts with different promoters and different expression methods. Feng et al. have successfully achieved the heterologous expression of VP28 protein with green alga *D. salina* and cyanobacteria *Anabaena* sp., and have explored the ability of the transgenic microalgae vaccine against white spot syndrome virus (WSSV). The results showed that genetically modified microalgae can effectively improve disease resistance and delay the death of shrimp (Feng et al., 2014; Jia et al., 2016). Subsequently, Zhai et al. (2019) highly expressed the VP28 protein in *Synechococcus* sp., and the expression efficiency was three times higher than that in *Anabaena*. In their recent research, *Synechocystis* PCC6803 was successfully carried out heterologous expression of VP28 protein. Oral transgenic *Synechocystis* PCC6803 can increase the enzyme activities in immune system and enhance the defense ability of WSSV infection in juvenile prawn. And these VP28 transgenic algae cells can be directly fed as juvenile shrimp bait without extraction and purification, which are expected to be applied to the aquaculture industry on a large scale. Somchai et al. (2016) constructed a shrimp yellow head virus (YHV) RNAi vector in the *C. reinhardtii* system, which improved survival by 22% after oral administration to prawn larvae. In the same way, Charoonnart et al. (2019) engineered the chloroplast genome of *C. reinhardtii* to express double-stranded RNA (dsRNA) designed to knock down key viral genes, Shrimps fed with dsRNA-expressed algal cells prior to YHV infection had 50% survival at 8 day-post infection, whereas only 15.9% survival rate was observed in control groups, and RT-PCR results revealed a lower infection rate in dsRNA-expressing algae treated shrimp compared to control groups. In all cases, aquatic animals were direct protected by feeding genetic modified microalgae. The above research and explorations give the direction for the development and large-scale application of genetically modified microalgae recombinant vaccines in the aquaculture field (Table 1).

**TABLE 1:** Research on developing genetically modified microalgae vaccine in aquaculture.

**Number**	**Pathogen**	**Antibacterial peptides (AP) Antigen (Ag)**	**Expression host**	**Vector**	**Promoter**	**Position**	**Expression (yield)**	**Protection Object**	**Effect**	**References**
1	*R. salmoninarum*	Ag:p57	*C. reinhardtii* [CC744]	/	/	Chloroplast and plasma membrane	/	*Oncorhynchus mykiss*	Produced p57-specific immunoglobulins (IgM) in different tissues	Siripornadulsil et al., 2007
2	*A. salmonicida*	Ag:AcrV, VapA	*C. reinhardtii* [WT, FUD50, FUD7]	pGA4	psaA, atpA psbD, psbA	Chloroplast	AcrV: 0.8% TP VapA: 0.3% TP	/	/	Michelet et al., 2011
3	WSSV	Ag:VP28	*C. reinhardtii* [CC741 mt+, Fud7 mt−]	pBA155 pSR229	psbA, atpA, psbD	Chloroplast	0.1–10.5% TSP	Shrimp	/	Surzycki et al., 2009
4	WSSV	Ag:VP28	*D. salina* [UTEX-1644]	pUX-GUS	Ubil	Chloroplast	78 mg/100 mL culture	Shrimp	59% Protection rate	Feng et al., 2014
5	WSSV	Ag:VP28	*Anabaena* sp. [PCC 7120]	pRL-489	psbA	Cytoplasm	34.5 mg/L culture Expression efficiency: 1.03% (dry weight)	Shrimp	68% Protection rate	Jia et al., 2016
6	WSSV	Ag:VP28	*Synechocystis* sp. [PCC 6803]	pRL-489	psbA	Cytoplasm	/	Shrimp	88.42% Relative survival	Zhai et al., 2019
7	WSSV	Ag:VP28	*C. reinhardtii* [TN72]	pASapI	atpA	Chloroplast	detectable	Shrimp	87% Relative survival	Kiataramgul et al., 2020
8	WSSV	Ag:VP19, VP28	*Synechococcus* sp. [PCC 7942]	pRL-489	psbA	Cytoplasm	vp19, vp28, vp (19 + 28) 5.0, 4.7, and 4.2%, (dry weight)	Shrimp	Activity of PO, SOD, CAT, and LYZ changed	Zhu et al., 2020
9	YHV	dsRNA-YHV(RNA)	*C. reinhardtii* [CC503 cw92mt+]	pSL18	psbD	Nucleus	41 ng/100 mL(1 × 10^8^ cells)	Shrimp	Increasing 22% protection	Somchai et al., 2016
10	YHV	dsRNA-YHV(RNA)	*C. reinhardtii* [*CC-5168*]	pSRSapI	psaA	Chloroplast	16.0 ± 0.9 ng dsRNA/L late-log phase culture	*Penaeus vannamei* Shrimp	50% survival at 8 day-post infection	Charoonnart et al., 2019

In addition, many international patents about recombinant microalgae vaccines for the prevention and control of aquatic diseases have been published or authorized (Table 2). This indicates that the theoretical research of microalgae vaccines is also extending to practical commercial applications. *Beta-nodavirusare* causes severe diseases, such as viral nervous necrosis (VNN) or viral encephalopathy and retinopathy (VER), which is detrimental to the growth and reproduction of marine fish, especially their larvae. In a patent filed by TransAlgae lnc. in Israel (Chen, 2016), the researchers selected Nervous Necrosis Viruses (NNV) capsid protein or fragment as an antigen to be expressed in microalgae subcellular compartment. The recombinant protein was delivered into the mucosal immune system of white grouper *Eyineyhelus aeneus* or the European sea bass *Dicentrarchus labrax* juvenile fish. The experimental results showed that the exogenous antigens could be presented successfully and stimulate the specific immune response in the fish body, so that transgenic microalgae could improve the survival rate of juvenile fish. At present, the company has been developing pipeline products for a fish disease and two major shrimp’s diseases in the aquaculture industry. In a patent filed by Durvasula and Durvasula (2008), the researchers introduced a DNA fragment encoding the antigen into a variety of microalgae, and the exogenous DNA fragment encoding product containing one or more key epitopes of the pathogen, such as WSSV, *V. harveyi*, etc. In one embodiment, the transgenic *D. salina* was initially fed to Artemia, and then the latter was used to feed shrimp larvae. In this method, the antigen molecule was presented indirectly to shrimp and successfully induced antibody within the gut tissue. Therefore, the oral administration of recombinant microalgae step by step is a measure conducive to the safety of commercial aquaculture. Similarly, in the patent applied by Tsai and Li (2011), one of the preferred examples used *Nannochloropsis* as an expression vector to express the foreign gene-encoded products, including rYGH of *Acanthopagrus latus*, Bovine lactoferricin (LFB), and capsid protein of WSSV (VP28). They, respectively, demonstrated that recombinant microalgae had the effects of the promotion of growth, the resistance to pathogens, and the resistance to viruses for the aquatic animals. The patent filed by the Ohio State University showed that the antigen can be delivered to the host through oral vaccination with transgenic microalgae and successfully induced immune response (Sayre et al., 2017). In one preferred embodiment, the successful expression of the foreign protein p57 can be detected from the fish mucus.

**TABLE 2:** Recent patents related to recombinant microalgae vaccine in aquaculture.

**Number**	**Patent Number**	**Pathogen**	**Antibacterial peptides (AP) Antigen (Ag)**	**Expression host**	**Vector**	**Promoter**	**Position**	**Effect**	**References**
1	US2016346373-A1	*Beta-nodavirus*	Nervous Necrosis Virus (NNV) Capsid protein or fragment thereof	*P. tricornutum*	pPhaT1	fcpA, fcp B	Vacuole	Improving the effectiveness of oral vaccines	Chen, 2016
2	WO2008027235-A1	WSSV	/	*D. salina*	pRrMDWK	Constitutive Inducible	Cytoplasm	Improving survival after virus attack	Durvasula and Durvasula, 2008
3	US2011014708-A1	/	LFB VP28	*N. oculata*	pCB740 pGEM-T	RBCS2, HSP70A	Nucleus Cytoplasm	Improving antibacterial and antiviral activity	Tsai and Li, 2011
4	US2017202940-A1	*R. salmoninarum*	P57 protein	*C. reinhardtii*	pUC18 pSSRC7	psbA β2-tubulin	Nucleus Chloroplast	Detecting antibodies *in vivo*	Sayre et al., 2017
5	US2014170181-A1	WSSV	VP28	*Chlorella* sp.	pGA4	/	Cytoplasm	100% protection rate	Moi et al., 2014

Recently, a number of researches on the application of genetically modified microalgae in aquaculture and the protection of intellectual property rights have been emphasized (Tables 1, 2). It is worth noting that many emerging algae companies (TransAlgae, Microsynbiotix, and Triton Algae) embarked on this trend and developed genetically modified organism (GMO) methods. In the current cases, oral administration leads to significant protective immunity and survival rate of orally vaccine animals. Oral delivery of antigens to aquatic organisms with microalgae can protect the antigen molecule from the degradation of metabolic digestive system. However, the detailed mechanism of antigen molecular presentation has not been dissected. The research on the direct or indirect oral feeding of recombinant microalgae for aquatic organisms is expected to better prevent, ameliorate, or treat diseases or disorders of aquatic animals in the aquaculture industry.

### Resistance of Microalgae Oral Agent to Polymicrobial Diseases

Various microorganisms such as bacteria, fungi, viruses, and even parasites infect the same biological host in different combinations. This process that causes acute or chronic diseases is called polymicrobial infection (Brogden et al., 2005). In the field of aquaculture, polymicrobial infection is a long-term and common phenomenon. Currently, the strategy for developing oral agents against polymicrobial infections using microalgae as a platform is mainly to heterologously express antimicrobial peptides. LFB is an antimicrobial peptide that can kill or inactivate many pathogens (Tsai and Li, 2011). Li and Tsai (2009) used the *Nannochloropsis oculata* as a host, expressed a broad-spectrum antibacterial peptide bovine lactoferricin (LFB), which has explored the antibacterial ability *in vitro* and *in vivo*. The experimental result showed that medaka fish fed with LFB-containing transgenic microalgae would have bactericidal defense against Vibrio *parahaemolyticus* infection in its digestive tract. After being infected by *V. parahaemolyticus*, the survival rate of the experimental group fed with transgenic microalgae increased to 85%. He et al. (2018) introduced the heterozygous antimicrobial peptide gene (Scy-hepc) into *Chlorella* sp., and evaluated the antibacterial effect of transgenic microalgae *in vitro* and *in vivo*. It was found that the extract of transgenic microalgae had antibacterial ability to the experimental bacteria. *In vivo* experiments, the relative survival rates of the *Sparus macrocephalus* and hybrid grouper fed with transgenic *Chlorella* after infected by *A. hydrophila* were 80 and 55%, respectively. Overall, the above examples provide some ideas for the development of a polymicrobial targeted recombinant microalgae vaccine in the aquaculture industry.

## Prospect of Algal-Based Oral Recombinant Vaccines

In fact, algae cells are known as the natural green factories. They have the advantages of high photosynthesis efficiency, direct use of solar energy, and CO_2_ fixation ability. Through genetic manipulation, a series of high value-added products such as pharmaceutical proteins, functional enzyme, and food additives can be efficiently expressed, which has unparalleled advantages (Georgianna and Mayfield, 2012; Dyo and Purton, 2018). The specific attributes and limitations of each system should be evaluated before selecting the most suitable oral vaccine development platform (Table 3). Compared with bacterial expression systems, eukaryotic microalgae can complete complex protein folding and modification to form active proteins that meet people’s specific needs for antigens and antibodies. Compared with yeast expression systems, microalgae can photosynthesize, sequestrate carbon, and reduce greenhouse gas emissions. Compared with genetically modified higher plants, it has a shorter culture cycle and is less restricted by seasonal weather conditions. Compared with the mammalian cell culture, it has lower production costs and can be easily scaled up. In addition, the microalgae-derived protein is biocompatible and can be directly consumed by animals without isolation and purification, thereby avoiding the cost of purification and extraction (Leon-Banares et al., 2004). It is important to stress that microalgae and cyanobacteria are extremely diverse with 100,000s of different species spread across the tree of life. Nowadays, a handful of these are recognized as edible (e.g., have GRAS status) and are used as food or feed components, which can serve as the cell factory to synthesize valuable products and oral delivery vehicle for subunit vaccines at the same time (Rosales-Mendoza et al., 2016). Therefore, it is completely feasible to apply algae cell metabolic engineering and synthetic biology research to the development of aquatic vaccines.

**TABLE 3:** Comparison of several systems used in oral vaccine development.

**Organism**	**Diversity of genetic tools**	**Growth rate**	**Modification capacity**	**Cultivation cost**	**Biosecurity**
Bacteria	++++	++++	+	+	++
Yeast	++++	+++	+++	+	+++
Mammalian cell	+++	+++	++++	++++	++++
Higher plant	+++	+	+++	++	++++
Microalgae	++	+++	+++	+	++++

At present, a variety of aquatic vaccines have been developed (Plant and Lapatra, 2011; Dadar et al., 2016). However, most of the vaccines usually need to be purified or need to be packaged with adjuvants. High cost and complicated processes (e.g., most of them require immunization by injection) have limited their application in large-scale aquaculture. Microalgae can be used as bait feed additive for aquatic animals in the form of living cells or powder, or made into granular bait as an additive, which generally has the effects of promoting growth, enhancing resistance, improving larval survival and body color for a variety of aquatic animals. At the same time, aquatic animals can obtain considerable immunity by oral administration, avoiding the physical damage caused to animals by injection or immersion immunization, reducing the operating cost and the burden on animals themselves. Therefore, if the antigen is highly expressed in the microalgae cells and the transgenic microalgae can be consumed as oral vaccine, it is expected to play an important role in the prevention and control of aquatic diseases (Mutoloki et al., 2015). As a feed for aquatic animals, algae cells can be used as a carrier to present heterologous expressed antigens which not only supplement nutrition but also play the role of vaccine.

Microalgae meet the sustainable development needs of multiple industries due to its unique advantages. However, there are still some problems with the microalgae expression system, such as low expression efficiency and poor stability, low recombinant protein content, immature genetic platform, etc. In addition, the situation of polymicrobial co-infection in aquaculture is becoming increasingly severe, whereas at present, there are no effective prevention and treatment measures for multi-pathogen infections, and related research is relatively scarce. In the future, with the development of oral vaccines using microalgae as a carrier, broad-spectrum antibacterial activity and multivalent recombinant microalgae vaccines are expected to flourish.

## Author Contributions

JF contributed to conceptualization and supervision. HW contributed to conceptualization, project administration, and supervision. KM and QB contributed to investigation, writing—original draft, review, and editing. YW, SC, and SZ contributed to writing—original draft. All the authors listed have approved the manuscript and agreed to authorship and submission of the manuscript for peer review.

## Conflict of Interest

The authors declare that the research was conducted in the absence of any commercial or financial relationships that could be construed as a potential conflict of interest.
